# Expression of E-cadherin, *α*-catenin, and *β*-catenin in the process of lymph node metastasis in oral squamous cell carcinoma

**DOI:** 10.1038/sj.bjc.6601124

**Published:** 2003-07-29

**Authors:** N Tanaka, T Odajima, K Ogi, T Ikeda, M Satoh

**Affiliations:** 1 Department of Oral Surgery, Sapporo Medical University School of Medicine, South 1, West 16, Chuou-ku Sapporo 060-0061 Japan; 2 Department of Clinical Pathology, Sapporo Medical University Hospital, South 1, West 16, Chuou-ku Sapporo 060-0061 Japan

**Keywords:** E-cadherin, *α*-catenin, *β*-catenin, oral squamous cell carcinoma, metastasis

## Abstract

Regional lymph node metastasis is a very important prognostic indicator. In the metastatic process, reduction in cell to cell adhesion including E-cadherin–catenin cell adhesion complex is an essential step. We investigated immunohistochemical expression of E-cadherin, *α*-catenin and *β*-catenin in 159 tissue samples from patients with oral squamous cell carcinoma and examined the correlation between their expressions and the presence of regional lymph node metastasis. Significantly greater reduction in expression levels of E-cadherin, *α*-catenin and *β*-catenin was found in the metastatic group (*n*=64) compared to the nonmetastatic group (*n*=95) (*P*=0.007, 0.001, 0.001, respectively). However, there was no significant correlation between their expressions and the features of the regional metastasis, the number of metastatic lymph nodes or the presence of extracapsular metastasis. These data suggest that evaluation of the immunohistochemical expression of E-cadherin, *α*-catenin and *β*-catenin is extremely valuable for the diagnosis of metastatic occurrence.

Among oral malignancies, squamous cell carcinoma is the most frequent and a number of studies have been conducted on the relationship between its clinical and pathological findings in order to improve the accuracy of prognosis for patients with this disease. We have already reported that a significantly reduced 5-year cumulative survival rate was observed in patients with oral squamous cell carcinoma and lymph node metastasis as compared with those without metastasis (Tanaka *et al*, 2001) and Jones *et al* (1993) also emphasised that lymph node metastasis is the most important prognostic indicator. As regards therapy, it is thus extremely important to identify the tumours that are likely to develop lymph node metastasis.

For metastasis to occur, cancer cells have to detach from the primary lesion as an initial step. E-cadherin is a Ca^2+^-dependent intercellular adhesion molecule in epithelial cells, which play an important role in establishing and maintaining intercellular connections and morphogenesis (Takeichi, 1991). The cytoplasmic terminus of the E-cadherin molecule has been shown to be linked to the actin cytoskeleton via *α*-catenin and *β*-catenin (McCrea *et al*, 1991; Ozawa and Kemler, 1992; Knusden *et al*, 1995), and E-cadherin is directly associated with *β*-catenin and indirectly linked via the *α*-catenin/*β*-catenin heterodimeric complex to *α*-catenin (Alberle *et al*, 1994; Oyama *et al*, 1994; Stappert and Kemler, 1994). It is reported that downregulation of the *α*-catenin, and *β*-catenin seems to be associated with dysfunction of E-cadherin-mediated cell adhesion and an increase in the metastatic potential of cancer cells (Kadowaki *et al*, 1994; Matsui *et al*, 1994; Ochiai *et al*, 1994; Pierceall *et al*, 1995; Rimm *et al*, 1995). Changes or alteration in the function and expression of the cell to cell adhesion molecule, E-cadherin, *α*-catenin and *β*-catenin, have been postulated to be an early event in the multiple process of tumour metastasis and an important factor in tumour progression (Birchmeier *et al*, 1993; Ponta *et al*, 1994). A variety of studies regarding the relationship between the expression of E-cadherin, *α*-catenin, *β*-catenin and the existence of lymph node metastasis in human cancers have been performed and some authors found a significant correlation (Sato *et al*, 1999; Zheng *et al*, 1999), while others did not (Mattijssen *et al*, 1993; Andrews *et al*, 1997).

This study was performed to clarify whether the expression of E-cadherin, *α*-catenin and *β*-catenin was correlated with the existence of lymph node metastasis in 159 patients with oral squamous cell carcinoma. To the best of our knowledge, this study is the first report that has examined the effect of altered expression of these proteins on regional metastasis in oral squamous cell carcinoma.

## MATERIALS AND METHODS

### Samples

We obtained 159 primary tumour samples, at the time of either biopsy for initial diagnosis or surgical resection, from 159 patients with oral squamous cell carcinoma consisting of 84 cases of carcinoma of the tongue, seven of the upper gingiva, 26 of the lower gingiva, 27 of the floor of the mouth, 13 of the buccal mucosa and two cases of carcinoma of the lip. The patients were treated at the Department of Oral Surgery, Sapporo Medical University School of Medicine. The treatment modality employed was surgery under snap-frozen section control or induction chemotherapy followed by definitive surgery. None of the samples examined was influenced by chemotherapeutic agents because in the cases in which such agents were used, the samples were obtained as biopsy before the chemotherapy. The age of the patients ranged from 26 to 85 years (average, 59.0 years). There were 55 male and 104 female patients. The grade of histological differentiation was determined following WHO 1997 criteria. The mode of invasion at the tumour–host borderline was classified into three types (expansive, moderately invasive and markedly invasive), with a modification of criteria used by Umeda *et al* (1992). Briefly, the expansive type had a well-defined borderline, the moderately invasive type exhibited no distinct borderline and had parenchyma consisting of large tumour cell nests and the markedly invasive type spread in small aggregates with ramifications or invaded diffusely without forming nests of tumour cells. We divided the samples into two groups: (1) pN(−) group (*n*=95): cervical lymph node metastasis was not recognised clinicopathologically for at least 2 years after surgery, (2) pN(+) group (*n*=64): cervical lymph node was recognised. Among the pN(+) group, radical neck dissection was performed in 30 cases, functional neck dissection in 22 cases and supraomohyoid neck dissection was performed in 12 cases.

### Immunohistochemistry

The tissue samples obtained were fixed in 10% buffered formalin and embedded in paraffin. Paraffin sections (6 *μ*m) were made, dewaxed, hydrated and immersed in citrate buffer (0.001 M citric acid, pH 6.0) for 5 min in an autoclave (121°C) for antigen retrieval. The sections were rinsed with 0.001 M phosphate-buffered saline (PBS) three times for 5 min each time, and then immersed in 0.3% hydrogen peroxide containing methanol to inactive endogenous peroxidase, and to block for nonstaining in normal goat serum in PBS for 30 min at room temperature. The sections were then incubated with primary antibody (anti-E-cadherin dilution with 1 : 100 (clone 36, Transduction Laboratories, Kentucky, USA) anti-*α*-catenin dilution with 1 : 100 (clone 5, Transduction Laboratories, Kentucky, USA) and anti-*β*-catenin dilution with 1 : 100 (clone 14, Transduction Laboratories, Kentucky, USA)) overnight at 4°C, rinsed with PBS three times for 5 min each time, and incubated with the secondary antibody, biotinylated goat antimouse immunoglobulin (IgG) (SC-2039; Santa Cruz Biotechnology, Santa Cruz, CA) for 30 min at room temperature and then rinsed again as previously described. The sections were finally incubated in avidin–biotin complex (Vector Laboratories, Burlingame, CA, USA) for 60 min at room temperature, then rinsed again as previously described. Visualisation of the peroxidase was performed using diaminobenzine in PBS with 0.005% hydrogen peroxide for 5 min. The sections were counterstained with haematoxylin, dehydrated and mounted. In each experiment, primary monoclonal antibodies were replaced with PBS in duplicate sections for negative controls.

### Evaluation of E-cadherin and catenins expression

All sections were examined by three independent pathologists who were blinded to the patients' clinical information, using a slight modification of the scoring method described previously (Andrews *et al*, 1997), and the staining pattern was evaluated semiquantitatively by comparing the intensity and cellular localisation of immunostaining with those of the adjacent normal epithelium as an internal positive control. If immunoreaction was uniformly membraneous and strong, similar to that in the internal controls, the staining was defined as normal. When immunoreaction was negative or weak, with either heterogeneous (mixed areas of positive and negative cells with normal membraneous staining) and/or altered cellular distribution (cytoplasmic and/or nuclear staining in more than 20% of the tumour cells, regardless of membraneous staining) of immunostaining, the staining was classified as abnormal. For each molecule, levels of immunoreaction were graded according to whether more than 75, 25–75 or less than 25% of cells showed the normal pattern of strong membraneous staining. For practical and statistical purposes, the immunoreactions in more than 75% and in less than 75% were also estimated as preserved and reduced, respectively. In tumours showing heterogeneity of staining, the immunostaining of the tumour was judged according to the prominent pattern. For each section, at least 500 cells, and usually more than 1000 tumour cells were analysed in 10 random high power fields (× 400). Whenever there were some discrepancies in the evaluation between observers, a consensus was reached using a multiheaded microscope. Cases in which internal positive control failed to show clear staining were excluded from this study.

### Statistical analysis

All statistical analyses were performed using SPSS 10.0J for Windows. The association between the presence of regional metastasis and clinicopathological variables was analysed using *χ*^2^ tests and Fishers exact tests. *P*-values <0.05 were considered to be statistically significant. Differences in survival analysed by the Kaplan–Meier method were assessed by the log-rank test.

## RESULTS

**Figure 1:**
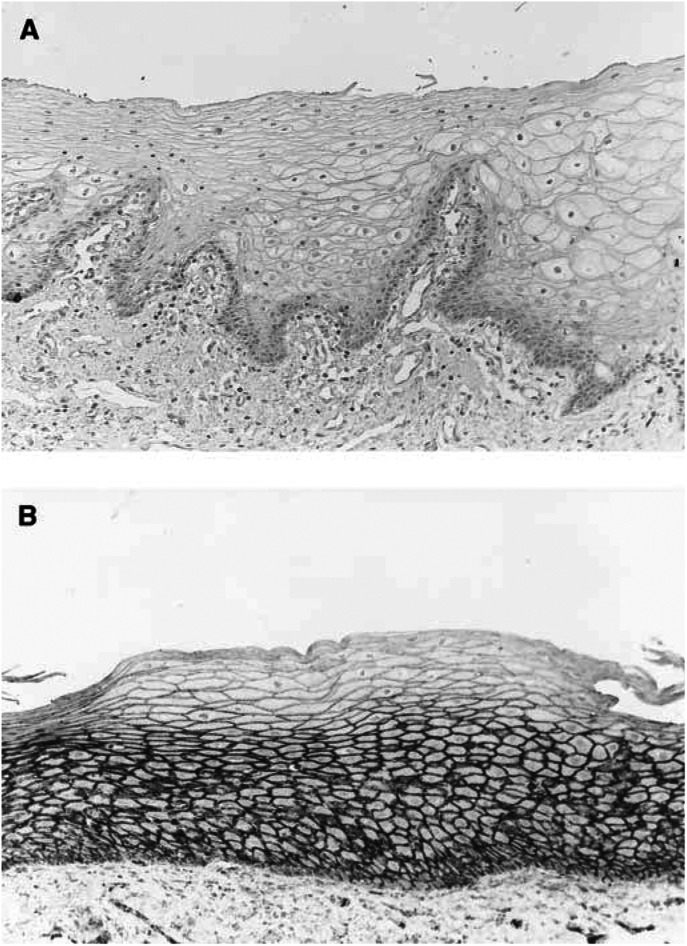
E-cadherin expression in normal oral epithelium. (**A**) Negative control, original magnification × 170; (**B**) E-cadherin is detected mainly on the cell membrane of the basal layer to the spinosum layer, original magnification × 170.

**Figure 2:**
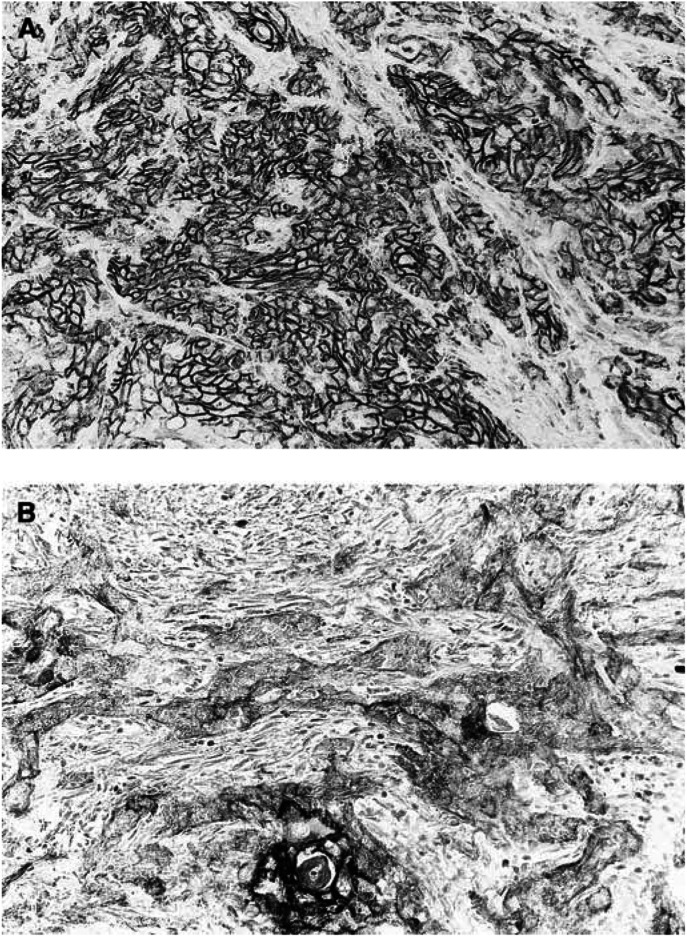
E-cadherin expression in oral squamous cell carcinoma. (**A**) An oral squamous cell carcinoma shows preserved E-cadherin expression, original magnification × 170; (**B**) An oral squamous cell carcinoma shows reduced E-cadherin expression, original magnification × 170.

Cytomembraneous expressions of E-cadherin, *α*-catenin and *β*-catenin were observed from the basal layer to the spinosum layer of normal oral epithelia and lost in the superficial layer (Figure 1

). In the cancer cells, the expression was detected only in the cytomembrane, or both in the cytoplasm and the nucleus. Representative immunohistochemical stainings of these molecules are shown in Figures 2

, 3

**Figure 3:**
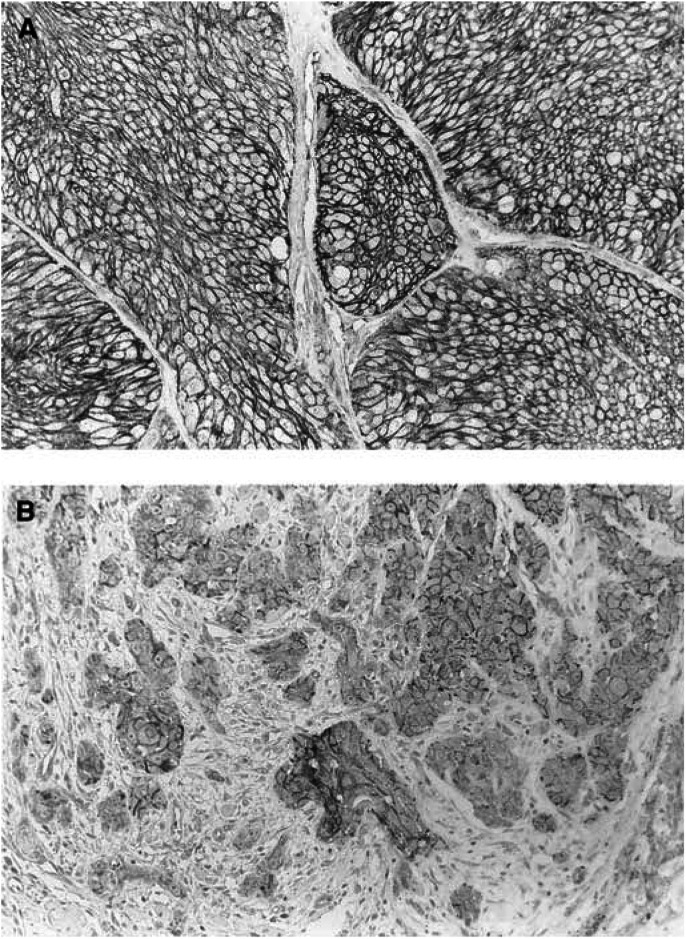
*α*-catenin expression in oral squamous cell carcinoma. (**A**) An oral squamous cell carcinoma shows preserved *α*-catenin expression, original magnification × 170; (**B**) An oral squamous cell carcinoma shows reduced *α*-catenin expression, original magnification × 170.

and 4

**Figure 4:**
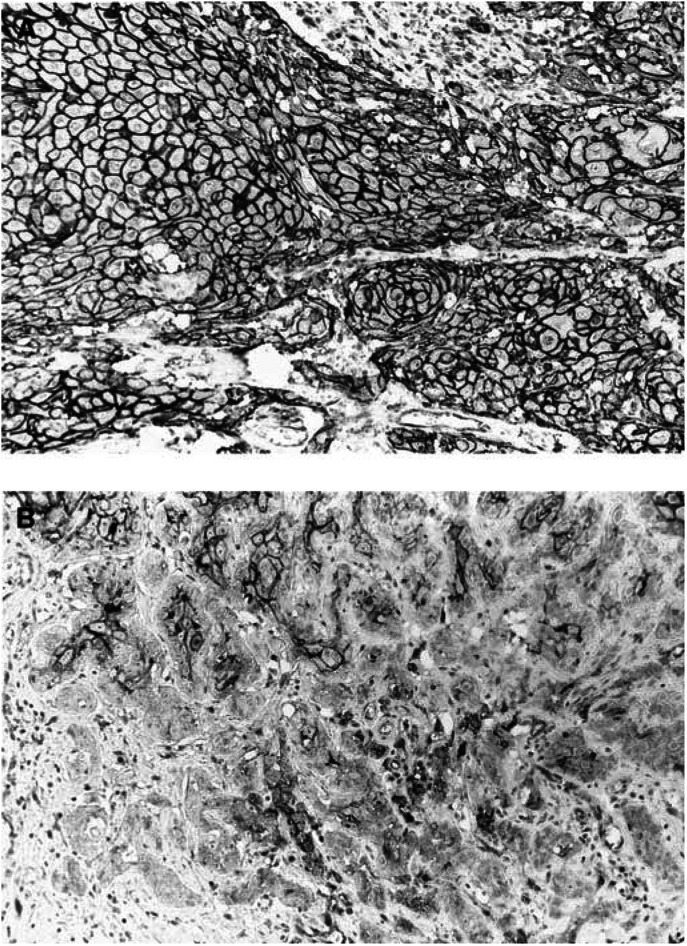
*β*-catenin expression in oral squamous cell carcinoma. (**A**) An oral squamous cell carcinoma shows preserved *β*-catenin expression, original magnification × 170; (**B**) An oral squamous cell carcinoma shows reduced *β*-catenin expression, original magnification × 170.

.

The expressions of E-cadherin, *α*-catenin and *β*-catenin are summarised in Table 1

**Table 1:** Correlation of regional metastasis and clinicopathological findings in 159 patients with oral squamous cell carcinoma

**Clinicopathological feature**	**pN(−)**	**pN(+)**	
*Age* (years)			
<50	18	11	*P*=0.914
50–69	61	44	*χ*^2^=0.830
>69	16	9	
			
*Gender*			
Male	38	17	*P*=0.057
Female	57	47	*χ*^2^=0.081
			
*Tumour site*			
Tongue	56	28	*P*=0.058
Upper gingiva	6	1	*χ*^2^=0.016
Lower gingiva	16	10	
Floor of mouth	8	19	
Buccal mucosa	8	5	
Lip	1	1	
			
*Tumor size*			
T1&T2	84	43	*P*=0.001
T3&T4	11	21	*χ*^2^=0.001
			
*Differentiation*			
Well	52	32	*P*=0.190
Moderate	36	21	*χ*^2^=0.158
Poor	7	11	
			
*Mode of invasion*			
Expansive	20	5	*P*=0.004
Moderately invasive	50	30	*χ*^2^=0.014
Markedly invasive	25	29	
			
*E-cadherin*			
>75%	47	20	*P*=0.007
25–75%	32	23	*χ*^2^=0.027
<25%	16	21	
			
*α*-*Catenin*			
>75%	35	9	*P*=0.001
25–75%	37	27	*χ*^2^=0.003
<25%	23	28	
			
*β*-*Catenin*			
>75%	38	7	*P*<0.001
25–75%	33	27	*χ*^2^=0.000
<25%	24	30	
			
Total	95	64	

. Out of the total 159 cases, reduced expression of E-cadherin, *α*-catenin and *β*-catenin was found in 92 (57.9%), 115 (72.3%) and 114 (71.7%), respectively.

### Association between expression of E-cadherin, *α*-catenin, and *β*-catenin and existence of lymph node metastasis (Table 1)

There was no significant association between age, gender, site of tumour or histological differentiation and the existence of lymph node metastasis in patients with oral squamous cell carcinoma.

Lymph node metastasis was found more frequently in the cases with T3 or T4 tumour than in those with T1 or T2 tumour (*P*=0.001), and there was a significant association between a high grade of mode of carcinoma invasion of primary tumours and lymph node metastasis (*P*=0.004).

Reduced E-cadherin expression was observed in 48 out of the 95 pN(−) cases, while it was seen in 44 out of the 64 pN(+) cases. There was a significant association between reduction of E-cadherin in primary tumours and lymph node metastasis (*P*=0.007). As well as E-cadherin, the expressions of *α*-catenin and *β*-catenin were also significantly reduced in pN(+) cases as compared with in pN(−) cases (*P*=0.001, <0.001, respectively).

### Association between expression of E-cadherin, *α*-catenin and *β*-catenin and features of lymph node metastasis (Table 2)

**Table 2:** Correlation of clinicopathological findings and features of regional metastasis

	**Number of metastatic lymph nodes**			**Character of metastatic lymph nodes**		
**Clinicopathological feature**	**Below two**	**Over three**		**Extracapsular**	**Intracapusular**	
*Tumour site*						
Tongue	21	6	*P*=0.166	11	16	*P*=0.680
Gingiva	5	5		5	5	
Floor of mouth	7	6		5	8	
Buccal mucosa	2	1		2	1	
						
*Tumour size*						
T1 and T2	22	9	*P*=0.272	11	20	*P*=0.136
T3 and T4	13	9		12	10	
						
*Differentiation*						
Well	14	10	*P*=0.331	10	14	*P*=0.288
Moderate	15	4		12	7	
Poor	6	4		1	9	
						
*Mode of invasion*						
Expansive	4	1	*P*=0.692	3	2	*P*=0.083
Moderately invasive	16	9		14	11	
Markedly invasive	15	8		6	17	
						
*E-cadherin*						
>75%	8	3	*P*=0.633	7	4	*P*=0.085
25–75%	13	7		9	11	
<25%	14	8		7	15	
						
*α-Catenin*						
>75%	4	2	*P*=0.806	4	2	*P*=0.420
25–75%	17	8		10	15	
<25%	14	8		9	13	
						
*β-Catenin*						
>75%	2	1	*P*=0.383	2	1	*P*=0.559
25–75%	12	9		9	12	
<25%	21	8		12	17	
						
Total	35	18		23	30	

We were able to investigate the features of lymph node metastasis, such as the number of metastatic lymph nodes (below two *vs* over three) and the existence of extracapsular metastatic lymph node in 53 pN(+) cases. We investigated the correlation between the expression of E-cadherin, *α*-catenin and *β*-catenin, mode of carcinoma invasion, degree of differentiation, site of the tumour, and tumour size and the features of metastatic lymph node; however, no significant association was recognised.

### Association between expression of E-cadherin, *α*-catenin, *β*-catenin and clinicopathological features (Table 3)

**Table 3:** Correlation of the expression of E-cadherin, *α*-catenin, *β*-catenin and clinicopathological findings

	E-cadherin				**α-catenin**				**β-catenin**			
**Clinicopathological feature**	**>75%**	**25–75%**	**<25%**		**>75%**	**25–75%**	**<25%**		**>75%**	**25–75%**	**<25%**	
*Tumour size*												
T1 and T2	57	40	30	*P*=0.446	39	46	42	*P*=0.508	40	44	43	*P*=0.294
T3 and T4	10	15	7	χ^2^=0.234	5	18	9	*χ*^2^=0.089	5	16	11	*χ*^2^=0.144
												
*Differentiation*												
Well	40	27	17	*P*=0.090	25	34	25	*P*=0.377	29	25	30	*P*=0.487
Moderate	21	23	13	*χ*^2^=0.346	14	25	18	*χ*^2^=0.696	12	27	18	*χ*^2^=0.227
Poor	6	5	7		5	5	8		4	8	6	
												
*Mode of invasion*												
Expansive	15	8	2	*P*=0.033	10	9	6	*P*=0.018	12	11	2	*P*=0.007
Moderately invasive	34	25	21	*χ*^2^=0.149	23	36	21	*χ*^2^=0.122	24	23	33	*χ*^2^=0.003
Markedly invasive	18	22	14		11	19	24		9	26	19	

The expression of E-cadherin did not appear to differ significantly based on tumour size or the degree of differentiation of the primary tumour. The reduced expression of E-cadherin was found to be related to the grade of carcinoma invasion (*P*=0.033), meaning that the reduced expression was more frequently found in the tumours with a high grade of mode of carcinoma invasion than in tumours with a low grade of mode of invasion.

The same investigation was performed for *α*-catenin and *β*-catenin. There were significant associations between the expressions of *α*- and *β*-catenins and the grade of carcinoma invasion of the primary tumour (*P*=0.018, 0.007, respectively), meaning that the expressions of *α*-catenin and *β*-catenins were reduced in many of the cases with a high grade of mode of carcinoma invasion.

### Association between expression of E-cadherin, *α*-catenin, and *β*-catenin and survival of patients with oral squamous cell carcinoma (Figure 5)

**Figure 5:**
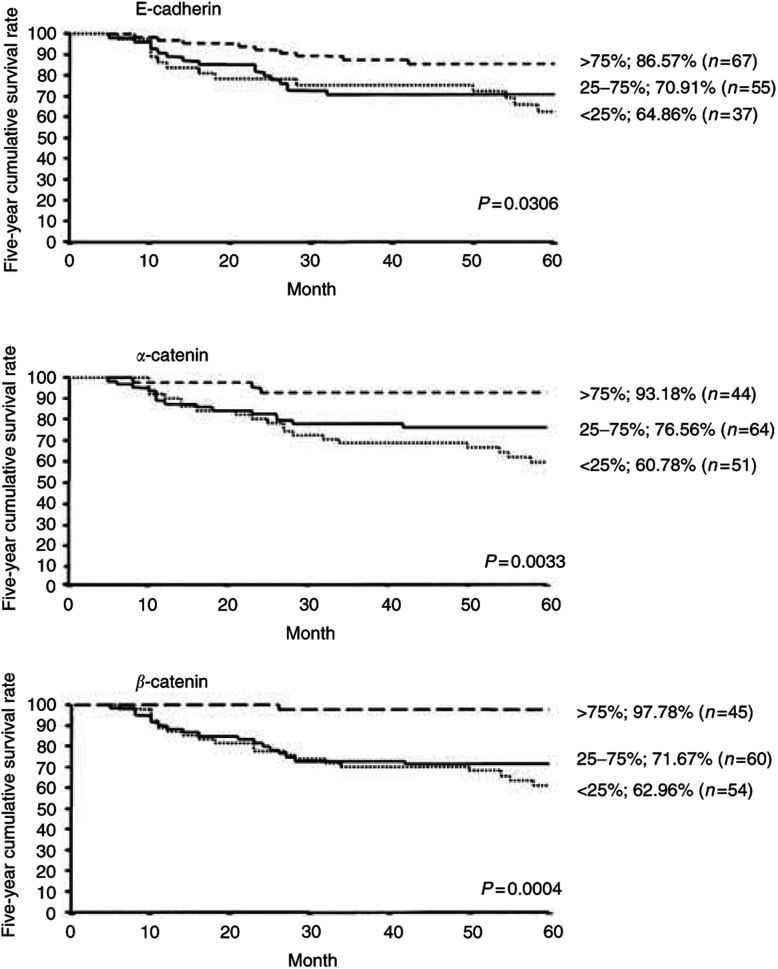
The Five-year cumulative survival rate (Kaplan–Meier). The 5-year cumulative survival rate of the patients with reduced expressions of E-cadherin or *α*-catenin or *β*-catenin tumours is significantly lower than that with preserved expressions of E-cadherin or *α*-catenin or *β*-catenin ones.

Survival in patients showing reduced expressions of E-cadherin, *α*-catenin and *β*-catenin was significantly shorter than in patients showing preserved expressions (*P*=0.0306, 0.0033, 0.0004, respectively).

## DISCUSSION

The incidence of neck metastasis in oral squamous cell carcinoma is relatively high (Lee and Krause, 1975, Martis *et al*, 1979).

The T classification of oral squamous cell carcinoma was correlated with the incidence of neck metastasis in the current study, which was consistent with the previous reports (Moore *et al*, 1986a, Mendenhall *et al*, 1988).

Moore *et al* (1986b) noted that tumour thickness was an accurate predictor of lymph node metastasis; however, its usefulness might be limited because measurement requires resection of the lesion.

The current study dealt with 159 oral squamous cell carcinomas, which is the largest series reporting the correlation between the expression of the E-cadherin-associated molecules and the presence of neck metastasis and it showed a significant correlation between them, indicating that the reduced expression of E-cadherin is a key function in the increased incidence of neck metastasis. Bukholm *et al* (1998) reported that there was no significant difference between the expression of E-cadherin and the presence of regional metastasis in human breast cancer, and it is said that the significance of changes in the E-cadherin complex may vary from tumour to tumour (Kinsella *et al*, 1993).

In the present study, there was a significant correlation between the expression of *α*-catenin or *β*-catenin and the presence of neck metastasis. It is reported that a significant association was seen between reduction in immunoreactivity of at least one of the following proteins: E-cadherin, *α*-catenin, *β*-catenin and *γ*-catenin and the presence of metastasis in breast carcinoma (Bukholm *et al*, 1998). Gofuku *et al* (1999) stated that reduction of *α*-catenin was a more sensitive and useful indicator than the reduction of E-cadherin in evaluating the potential for tumour invasion and metastasis in human colorectal cancer. However, the mechanism of E-cadherin-mediated intercellular adhesion is not fully understood yet and further study is needed in order to clarify which protein expression among E-cadherin, *α*-catenin, and *β*-catenin is most useful for diagnosis and prediction of metastasis and tumour invasion.

We performed a clinical examination of oral squamous cell carcinoma cases which received neck dissection and the results indicated that the prognoses of the cases which had three or more metastatic lymph nodes and/or extracapsular metastatic lesion were poor. Therefore, we investigated the correlation between the expression of E-cadherin, *α*-catenin and *β*-catenin and the presence of three or more metastatic lymph nodes and/or extracapsular metastatic lesions. However, there were no correlations between them. We reported that from ultrastuctural observation of primary and metastatic oral squamous cell carcinomas, morphologic similarity was recognised between the primary and metastatic lesions; however, the features of junctional complexes varied from the primary lesion to the metastatic one in some cases (Tanaka *et al*, 2002). Whether regional metastasis occurs or not may depend on the features of the primary lesion, including the intercellular adhesion of the tumour cells. However, the features of the metastatic lesion may be independent of those of the primary lesion and may be mainly influenced by circumustances unique to the metastatic lesion itself.

Mode of carcinoma invasion evaluated from the biopsied specimens has already been reported to be related to regional metastasis (Yamamoto *et al*, 1984), which is consistent with the result of the current study. Tumour invasion is also related to intercellular adhesion and there were also correlations between the mode of carcinoma invasion and the expressions of E-cadherin, *α*-catenin and *β*-catenin.

The T classification correlated with the presence of neck metastasis, however not with the expression of E-cadherin, *α*-catenin or *β*-catenin. Growth of the tumour may correlate not only with intercellular adhesion but also with other factors.

The correlation between mutation or methylation of E-cadherin, *α*-catenin or *β*-catenin and the presence of metastasis is now under investigation in order to clarify the mechanism of metastasis (Ogi *et al*, 2002).

In conclusion, in the cases with oral squamous cell carcinoma that develop regional metastasis, the expression of E-cadherin, *α*-catenin and *β*-catenin was reduced and therefore it is strongly suggested that immunohistochemical investigation of these proteins is presently of value for the purpose of diagnosing the presence of metastasis.
